# Herbivorous dietary selection shown by hawfinch (*Coccothraustes coccothraustes*) within mixed woodland habitats

**DOI:** 10.1098/rsos.230156

**Published:** 2023-05-10

**Authors:** Ewan H. Stenhouse, Paul Bellamy, Will Kirby, Ian P. Vaughan, William O. C. Symondson, Pablo Orozco-terWengel

**Affiliations:** ^1^ School of Biosciences, Cardiff University, Cardiff CF10 3AT, Wales, UK; ^2^ RSPB Centre for Conservation Science, The Lodge, Sandy SG19 2DL, UK

**Keywords:** dietary selectivity, hawfinch, metabarcoding, species management

## Abstract

Knowledge of diet and dietary selectivity is vital, especially for the conservation of declining species. Accurately obtaining this information, however, is difficult, especially if the study species feeds on a wide range of food items within heterogeneous and inaccessible environments, such as the tree canopy. Hawfinches (*Coccothraustes coccothraustes*), like many woodland birds, are declining for reasons that are unclear. We investigated the possible role that dietary selection may have in these declines in the UK. Here, we used a combination of high-throughput sequencing of 261 hawfinch faecal samples assessed against tree occurrence data from quadrats sampled in three hawfinch population strongholds in the UK to test for evidence of selective foraging. This revealed that hawfinches show selective feeding and consume certain tree genera disproportionally to availability. Positive selection was shown for beech (*Fagus*), cherry (*Prunus*), hornbeam (*Carpinus*), maples (*Acer*) and oak (*Quercus*), while Hawfinch avoided ash (*Fraxinus*), birch (*Betula*), chestnut (*Castanea*), fir (*Abies*), hazel (*Corylus*), rowan (*Sorbus*) and lime (*Tilia*). This approach provided detailed information on hawfinch dietary choice and may be used to predict the effects of changing food resources on other declining passerines populations in the future.

## 1. Introduction

To confidently identify the available and consumed food items within the environment of an animal remains a major challenge in ecology [1]. Accurate identification is especially difficult if the study species use a wide range of food resources within diverse environments [1–3]. Birds, like all organisms, must adapt to local habitats and resources in order to satisfy their energetic demands [4]. Individual birds must select which habitat or foraging areas to visit more frequently than others in order to fulfil their daily energy budget and dietary needs [5]. Food types are deemed more rewarding if they provide greater energy per handling time than alternative resources, with many species selecting mixed diets in order to meet energetic and nutritional demands [6,7].

To elucidate a species' dietary composition using traditional morphology-based methods can be time consuming, and biased towards identification of distinguishable and intact undigested or semi-digested dietary items [3]. Molecular techniques such as DNA metabarcoding are being used increasingly to assess the diet of a range of organisms [1,8–11]. These techniques require minimal *a priori* knowledge of the dietary composition of the study species [2,12,13], and a wide range of taxa can be identified to fine taxonomic levels [14–17]. However, quantitative metabarcoding outputs are still a point of discussion, as several factors can introduce bias into results and subsequently provide unreliable biomass estimates [18].

Despite birds being one of the best-studied animal classes, few studies have used molecular techniques to improve understanding of their trophic ecology [11,19,20]. In comparison with studies on mammals, in particular bats, the application of faecal metabarcoding within passerines studies is rare. Metabarcoding is an evolving field, with research being undertaken on an expanding number of passerine species [11,20,21]. However, previous studies have mainly focused on bird species with specialists diets and narrow feeding niches, while DNA metabarcoding has been seldom applied in studies focusing on more generalist species (but see [22]).

Partitioning of available resources has been highlighted as a key factor structuring bird communities [23]. Differences in morphological and physiological characteristics result in resource use and foraging strategies differing between species [23]. Additionally, as food availability is often strongly impacted by seasonality, birds can respond to fluctuating temporal and spatial availability of resources through adapting a specialized foraging behaviour [24]. Specialized foraging can reduce resource competition among individuals, a beneficial foraging strategy under strong intra-specific competition pressure [25]. While specialized foragers may benefit from improved foraging efficiency, they may be vulnerable to fluctuations in abundance of the limited resources exploited [26]. Therefore, the adaptive value of specialization may vary temporally and spatially, due to fluctuations in resource availability or level of competition [27,28].

It is important to note, however, that foraging can be a flexible activity. Optimal foraging theory states that resources are exploited which maximize net energy intake while minimizing energetic costs, through a trade-off between resource profitability and searching time [29]. Furthermore, the theory suggests that ‘specialized’ predators should adopt a more generalist feeding strategy when preferred foodstuffs are in low abundance, incorporating foodstuffs previously ignored [30]. Generalists can also show selectivity towards certain dietary items, and be more selective when preferred items are available in the environment [31]. Dietary plasticity therefore is an important mechanism enabling response to environmental changes such as seasonal or temporal fluctuations in resources, or anthropogenic pressure [32,33], with a suboptimal diet being detrimental to individual fitness [34].

Dietary preferences in birds, or the greater consumption of a certain food resource despite equal opportunity to feed on an alternative food [35], can be linked to physiological capabilities and nutritional requirements [36,37]. The process of ‘selection’, unlike preference, is where an animal makes a choice among differing resources and consumes them disproportionately to their availability [38]. This process is a result of interactions between dietary selection and a number of factors which modify them, including handling time, the spatial distribution of resources and availability of alternative resources [35,39,40]. Increasing our understanding of resource use in relation to food availability is a focal point within the study of bird communities [4] and may provide valuable insights into the mechanisms behind declines seen in some woodland passerines.

The hawfinch (*Coccothraustes coccothraustes*) breeds across the Palaearctic, with Britain being its westerly range limit [41]. Over recent decades, the number of hawfinches has declined substantially across Britain, with a 76% reduction in occupied 10 km squares between 1968 and 2011 [42]. Hawfinches persist in highly wooded landscapes containing mature and diverse tree assemblages [43]; however, there is a paucity of information regarding hawfinch diet, with the only information based upon visual observations [44]. Hawfinches are thought to be dietary specialists adapted to use large-seeded tree species due to their large and powerful beak [44]. During the breeding season (April–August), hawfinches were observed feeding most regularly on seeds of beech (*Fagus* sp.) and wych elm (*Ulmus glabra*), as well as the buds of cherry (*Prunus* sp.), hornbeam (*Carpinus betulus*) and larch (*Larix decidua*) [44]. Flowers from oak (*Quercus* sp.), beech and maples (*Acer* sp.) were also consumed [44]. However, this dietary information may in part reflect the relative ease of observing these food items, and there has been no advancement of hawfinch dietary knowledge since these visual observation studies. No studies have investigated whether hawfinches are actively selecting these food items.

This study combined results from DNA metabarcoding of hawfinch faecal samples with the relative abundance of naturally occurring tree genera from three hawfinch foraging areas within the UK: the Wye Valley, north Wales and the New Forest. We predicted that the prevalence of some taxa detected in the diet of hawfinches will be consumed disproportionately to their availability, indicating dietary selectivity or avoidance. The results from this study may then be used to inform woodland management strategies for hawfinch and other declining woodland passerines.

## 2. Materials and methods

### 2.1. Study areas

We used three distinct study areas, based on existing ringing studies within managed woodlands. The first study area incorporated a segment of the Wye Valley between Monmouth and Chepstow along the border of England and Wales. The second was near Dolgellau, Gwynedd in north Wales, with the third in the New Forest, Hampshire. The Wye Valley and north Wales study areas were similar in habitat type, consisting of steeply sloping valleys and heterogeneous, mature woodland dominated by beech, oak and ash (*Fraxinus* sp.). Other notable components of the landscape included farmland and conifer plantations. The New Forest study area was heterogeneous mature woodland dominated by oak, with an understorey flora comprising Holly (*Ilex aquifolium*) and bramble (*Rubus* sp.).

### 2.2. Collection of hawfinch faecal samples

From March to July 2017–2019, hawfinches were caught under licence using mist nets at artificial feeding stations within the study areas. The artificial feed sites used to attract hawfinches for capture have been operational for a number of years within regions of hawfinch population strongholds [43,45]. Sunflower seeds (*Helianthus* sp.) are provided between March and June at these artificial feeding sites when targeting hawfinches, to attract them down from the canopy for capture. Once caught, individual hawfinches were placed in new, clean paper bags to collect uncontaminated faecal samples. Faecal samples were stored at −20°C between 1 and 6 h after collection. A total of 57 additional samples had previously been collected and stored at −20°C during 2016–2017, in anticipation of this study.

### 2.3. DNA extraction and sequencing

Using the protocol for pathogen detection with modifications by [11,20], designed to improve DNA yields from avian faeces (electronic supplementary material S1), DNA was extracted from 261 faecal samples using the Qiagen QIAamp DNA Stool Mini Kit (Manchester, UK). The second internal transcribed spacer (ITS2) gene of the nuclear ribosomal DNA was targeted for amplification of plant DNA with primers modified to an identification barcode [46]. Library preparation for Illumina sequencing was undertaken via NEXTflex Rapid DNA-Seq kit (Bioo Scientific, Austin, USA) with unique adapters added. A MiSeq desktop sequencer was used for sequencing.

The bioinformatics pipeline followed [47]. In brief, reads were clustered to zero-radius operational taxonomic units (hereafter zOTUs), based on a 100% clustering threshold, resulting in high taxonomic resolution and preventing incorrect clustering of variants [46]. Tree species represented by multiple zOTUs were collapsed so multiple zOTUs were represented by a single entry. The ITS2 database [48] was used for comparison with known reference sequences. Sequences were assigned a taxonomic identity from the ITS2 database using a 97% identity threshold [48,49].

### 2.4. Tree surveys

For prevalence of different tree species, we used data collected during other studies within the same woods from long-term ongoing hawfinch projects [41,43]. A total of 280 nest sites and random locations across all three study areas were visited between 2013 and 2016 within the same woods [41]. A further 199 locations were visited in 2017 from the north Wales study area. These data were based on foraging locations from GPS tracking of individual hawfinches and random locations in any woodland within the north Wales study area [43]. As these samples of tree prevalence were based on hawfinch faecal samples collected from the same woodlands, they constitute a reasonable sample of the tree prevalence available to hawfinches in each study area.

At all locations a quadrat of 10 m × 10 m was marked out, with the given location at the southwest corner of the quadrat. Within each quadrat, trees were identified and circumference at breast height (CBH) of all trees and shrubs was recorded. As many closely related tree species are difficult to identify to species, with some genera frequently hybridizing, morphometrically similar species were recorded together (e.g. ‘oak’ includes *Quercus robur*, *Quercus petraea* and hybrids). Any trees which had a CBH less than 20 cm diameter were not recorded to discount saplings, which are known not to be used as a food resource by hawfinch. As all tree surveys were undertaken within managed woodlands, large trees were rare. Across all study areas, from 3316 trees measured, 0.24% were greater than 1 m diameter (greater than 314 cm CBH). Within north Wales and the Wye Valley, trees greater than 1 m made up 0.1% of all trees recorded (2296 and 930 trees measured, respectively). Within the New Forest, of the total number of trees measured (90), large trees contributed to 5.5%.

### 2.5. Statistical analysis

Tree survey count information was analysed in conjunction with plant dietary data produced from DNA metabarcoding of hawfinch diet to determine diet selectivity. Frequency of occurrence (FOO) of taxa was calculated by totalling the number of instances that a given taxon occurred across all hawfinch samples. This was then calculated as a percentage of the total number of samples (%FOO) by dividing the FOO value by the total number of hawfinch faecal samples collected (detailed in table 1). FOO was chosen, as DNA metabarcoding cannot accurately provide biomass measurements of dietary taxa [15,18]. All analyses were done at the genus level to standardize the taxonomic level of recording across the dietary and tree prevalence data.

**Table 1.** The FOO expressed as a percentage (%FOO) of tree genera represented in 261 hawfinch faecal samples across the UK that tested positive for herbivorous dietary items.

percentage of samples testing positive for herbivorous taxa (%FOO)				
genus	all (*n* = 261)	north Wales (*n* = 108)	Wye Valley (*n* = 134)	New Forest (*n* = 19)
*Ulmus*	16.5	3.7	29.1	0
*Tilia*	3.8	0.9	6.7	0
*Taxus*	5.7	4.6	6.7	5.3
*Sorbus*	1.9	3.7	0.7	0
*Salix*	4.6	10.2	0.7	0
*Quercus*	37.5	37.0	31.3	84.2
*Prunus*	23.8	27.8	22.4	0
*Pinus*	1.5	0.9	0	15.8
*Picea*	3.8	2.8	5.2	0
*Larix*	5.7	5.6	6.7	0
*Ilex*	7.7	4.6	3.7	52.6
*Fraxinus*	8.0	10.2	6.0	0
*Fagus*	66.3	47.2	83.6	52.6
*Cupressus*	0.8	0.9	0	0
*Crataegus*	0.4	0	0.7	0
*Corylus*	7.3	7.4	8.2	0
*Castanea*	0.8	0.9	0	0
*Carpinus*	19.9	30.6	0	0
*Betula*	11.1	15.7	5.2	26.3
*Alnus*	3.1	1.9	0	0
*Acer*	16.9	17.6	15.7	21.1
*Abies*	1.5	2.8	0.7	0

To determine spatial differences in hawfinch diet, tree genera determined by DNA metabarcoding were visualized using non-metric multi-dimensional scaling (nMDS) analysis via the function *metaMDS* in the *vegan* package [50] in R v. 3.6.3 [51]. The nMDS was performed with Jaccard dissimilarities in three-dimensional space (*k* = 3). The spider plot was produced using nMDS results via *ordispider* and plotted through ggplot2 [52]. Differences in tree communities between study areas were tested with multi-variate analysis of variance using the *adonis* function of the *vegan* package.

Hawfinch dietary choice may be greatly influenced by food availability. It is possible to elucidate feeding selectivity by testing the observed prevalence of taxa in the diet against the prevalence predicted by a null model that uses the relative abundance of taxa in the foraging environment. This enables us to differentiate between taxa which are consumed at greater, lesser or equal frequency than expected based upon their availability. Hawfinch dietary choices were analysed using null models within the *econullnetr* package [53], which simulated the expected frequencies with which different tree genera would be found across the faecal samples if hawfinches selected food items purely based on their relative abundance in the tree surveys (i.e. showing no selection).

Data were analysed by including all tree quadrats throughout the three study areas where hawfinch was known to be feeding and comparing this with the equivalent presence/absence molecular dietary data. Local-scale models were run separately using only tree survey and equivalent molecular dietary data from the individual study areas. Models were run for 999 iterations to produce frequency distributions of expected rates of herbivory based on the plant food available. Observed herbivory rates were then compared with those expected by chance. When the observed consumption rates fell outside the central 95% of simulated values, this indicated statistically significant deviations from random herbivory and indicated dietary selection. A total of six tree genera (*Euonymus*, *Hedera*, *Malus*, *Sambucus*, *Sequoia* and *Tsuga*) were excluded from the analysis as they were surveyed, but not detected in the diet. Artificially provided sunflower seed was also removed as this was not naturally available within the study areas. All analyses were done at the genus level to standardize the taxonomic level of analysis, as some closely related tree species could not be differentiated in the field.

## 3. Results

**Figure 1.**
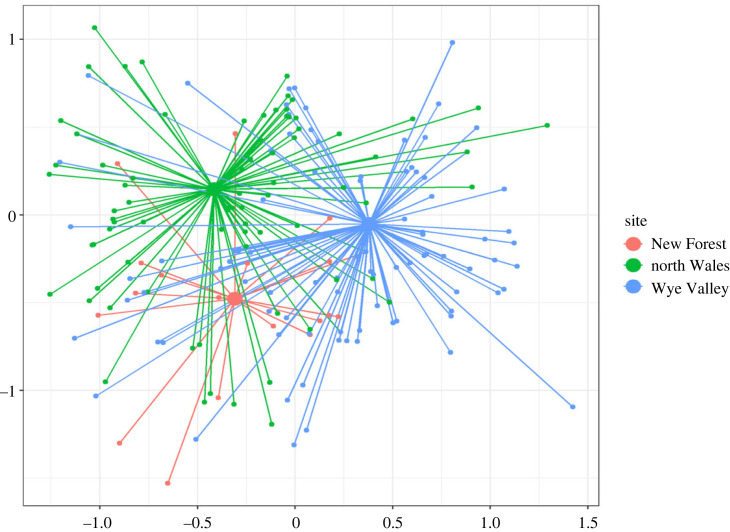
Spider plot showing dietary differences in herbivorous taxa consumed by hawfinch across the three sampling areas. Smaller nodes represent individual hawfinch with connecting lines joining the individual to the mean centroid (larger nodes) of its region. Stress = 0.12.

A total of 6 328 388 sequences were retrieved from 261 hawfinch faecal samples, while 193 610 sequences were detected within negative controls. A total of 202 849 unique sequences were removed due to contamination, tag-jumping and poor-quality sequences or reads likely to be a result of degradation. Dietary items most frequently detected in hawfinch diet were beech, hornbeam and oak, with FOO for all genera included in table 1. Dietary differences between hawfinch populations were visualized in figure 1.

Tree count data revealed a total of 27 tree genera across the three study sites. The most frequently recorded (number of recorded counts in brackets) were hazel (*Corylus*) (493), birch (*Betula*) (455) and oak (450). In north Wales, birch (78), ash (*Fraxinus*) (47) and oak (44) were the most recorded tree genera. The Wye Valley was dominated by ash (57), beech (55) and hazel (46), while the genera most frequently recorded within the New Forest were beech (7), holly (*Ilex*) (4) and oak (3). The *adonis* results revealed a significant difference in tree species composition between study area landscapes (*R*^2^ = 0.75, *p* = 0.01).

**Figure 2.**
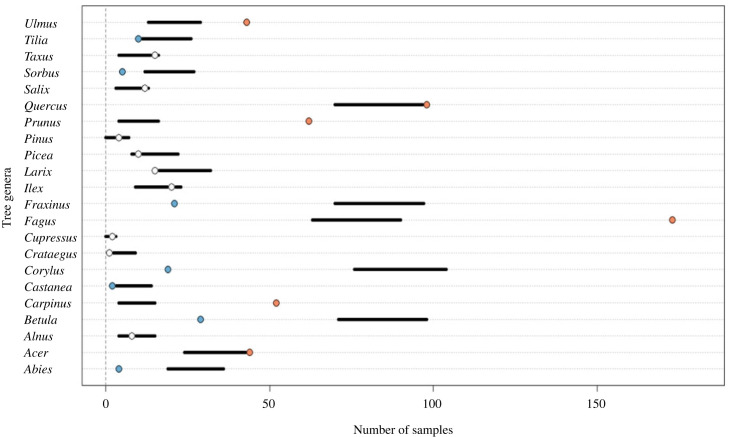
Dietary selectivity of hawfinches at a UK scale. Horizontal lines represent 95% confidence intervals for predicted FOO generated by the null model (the number of samples in which taxa would be present if consumed in proportion to their availability). White circles indicate tree genera eaten in proportion to their availability (i.e. falling within the 95% confidence interval); blue circles: genera eaten in lower proportions than expected; orange circles: genera eaten at a greater proportion than expected.

From a total of 261 hawfinch faecal samples, the resource selection model revealed that hawfinch showed feeding selectivity and avoidances (figure 2; electronic supplementary material, figure S2). Analysis of hawfinch populations at a national scale revealed selectivity for six genera: beech, cherry, elm, hornbeam, maples (*Acer*) and oak. Hawfinch was shown to avoid seven genera: ash, birch*,* chestnut (*Castanea*), fir, hazel, lime (*Tilia*) and rowan (*Sorbus*).

Analysis of feeding preferences from 108 hawfinch faecal samples in north Wales showed feeding selectivity for four genera: beech, cherry, hornbeam and yew (*Taxus*). Six genera were consumed less frequently than expected: ash, birch, elm, fir, hazel and rowan. Analysis of 134 faecal samples within the Wye Valley revealed selectivity for five genera: beech, elm, maples, oak and yew. Significantly weaker interactions than expected were revealed for ash, fir, hazel and lime. Conversely, 19 hawfinch faecal samples analysed from the New Forest showed significant dietary selectivity towards oak and did not show significant dietary avoidance for any genus.

## 4. Discussion

Hawfinches were found to strongly select for feeding on cherry at both national and landscape scales, which has previously been highlighted as a frequently used key food resource [44]. This selectivity may be due to the high nutritional value of cherry, or that hawfinch can handle cherry efficiently, giving a high energy reward per handling time [54]. The selectivity shown may also be consistent with optimal foraging theory, which suggests that when food is abundant, individuals are likely to be choosy and will select higher quality food [55]. Hawfinch is generally considered a habitat specialist; however, dietary composition and selectivity were shown to vary between sites at a relatively local geographical scale with similar foraging environments, as has been seen in the Daubenton's bat (*Myotis daubentonii*) [31,41]. Thus, hawfinch may show local adaptions to efficiently use certain tree genera in order to reduce the cost of associated foraging [31]. These results, combined with *a priori* knowledge, highlight the potential importance of certain tree genera in hawfinch persistence within their core habitat of mature semi-natural woodland [41]. Hawfinch may also be selectively feeding on cherry due to their morphological adaptations of having a large, powerful bill, permitting them to crack cherry stones, a food resource unavailable to many other bird species. Significant relationships between beak morphology and feeding ecology have been found in Darwin's finches, great tit (*Parus major*), shorebirds and raptors [56–58]. Local conditions can also result in morphological adaptations within a species, for example in birds, food type and feeding behaviour are strongly linked to bill shape and size [59,60]. This morphological adaptation and foraging specialization may allow hawfinch to coexist with other avian species within the same habitat (in this case broad-leaved woodlands) through niche-partitioning, therefore avoiding competitive exclusion [61].

Key food resources identified through DNA metabarcoding such as elm and hornbeam were also shown to be strongly selected for, strengthening the hypotheses in earlier studies that these tree species act as regularly used food resources [44,62]. The impact of Dutch elm disease which resulted in the estimated loss of 20 million elm trees in the UK [63] may have induced hawfinch to seek out and hence feed preferentially on elm trees that remain, especially if the loss of this resource may reduce hawfinches' ability to retain a suitable breeding condition [41]. Elm may be providing food resources such as buds and flowers during early spring, when availability of other resources is low [41].

Hawfinch showed dietary avoidance of ash. This genus has increased in abundance within broad-leaved woodland since the 1940s, making up 13.1% of total broad-leaved area in 2002 [64]. Ash has not been highlighted as a frequently used food resource [44], and the avoidance shown may be due to other more rewarding food resources (such as cherry) being available. Furthermore, ash seeds are known to contain phenolic compounds which may limit their consumption by hawfinch [65]. Throughout much of the UK ash trees are dying from ash dieback disease (*Hymenoscyphus fraxineus*) [66]. Although these trees form a major component of UK forests, our data suggest the loss of ash trees may not affect hawfinches. Hazel was also shown to be avoided by hawfinch, and this result supplements previous observational data showing hawfinch do not use hazel as a food resource [44,62]. Hazel is an understorey shrub species, and while hawfinch requires a complex understorey for persistence within woodland, they are not known to feed within the understorey layer [67]. Feeding occurs on the ground and in the canopy [44,62]. Additionally, the seeds of hazel are large, and as a result, the only avian species able to handle them are greater spotted woodpecker (*Dendrocopos major*) and nuthatch (*Sitta europaea*), due to their feeding behaviour of ‘hammering’ open the seed [68].

The dietary avoidance shown by hawfinch seen in this study may also be due to the presence of secondary compounds within certain dietary items [69]. The presence of toxic secondary compounds can decrease the value of the food resource [54,70]. Food which contains high levels of toxins is often less preferred and of lower quality, and generalist species will forage on a preferred higher quality food resource, incorporating the lower quality food into the diet only when the preferred food choice falls below a certain threshold [71]. The impacts of secondary compounds may be dependent on the amount consumed, rather than their concentration [72]. For example, bullfinches (*Pyrrhula pyrrhula*) are known to reject seeds containing high levels of phenols [65]. This study revealed rowan, known to contain secondary compounds [73], was strongly avoided by hawfinch based on its relative abundance. It is possible that the concentrations of toxins within rowan limit consumption, therefore when this toxin capacity is exceeded hawfinch switch to a different food resource.

It is important to take into consideration that seasonal diet expansion or switching may occur. This may be due to physiological processes such as gut modulation through the alteration of digestive physiology, allowing more efficient nutrient uptake [74]. Birds may switch or expand their dietary niche breadth in relation to increasing nutrient requirements for migration or breeding [75,76], or as a result of declining food availability [77]. The results from this study are only a temporal snapshot, and while focused within the spring and summer months, it can be assumed that during this sampling period hawfinch would have differing nutrient and energetic requirements than in autumn and winter. To capture a comprehensive temporal picture of hawfinch dietary selectivity, tree abundance and dietary data should be collected throughout the year. It is also important to consider the impact of masting, the synchronized occurrence of mass seed production within forests [78]. As the woodlands were largely managed for timber, large trees (greater than 1 m diameter) likely to have disproportionate food supply during flowering, or years of high seed set, were rare in our study areas. While these large trees could attract disproportionate numbers of hawfinches, it was not possible to look at these effects with our data.

While the methodology used in this study provides a broad overview of hawfinch dietary selection, there are limitations to this approach which should be considered. The detection of species abundance and distribution is frequently imperfect within ecological studies [79], due mainly to observer error or rarity of species [80,81]. While many tree surveys were undertaken, complete surveys of the woodlands were not possible due to the time needed to accomplish this. From the taxa shown to be selected for by hawfinch, only oak and beech were frequently encountered (450 and 399 counts, respectively). Cherry (41), elm (92), hornbeam (38) and maples (150) were scarce. Highly selected-for tree genera seen in this study may be a result of low measured abundance from the tree surveys, potentially due to the patchy distribution of certain tree genera within woodlands. This may result in skewed estimates of overall tree richness and abundance. Nonetheless, without the application of remote sensing data such as hyperspectral reflectance data, on-the-ground field quadrats were the most appropriate and effective sampling methodology available to quantify tree genera abundance.

DNA metabarcoding is unable to accurately provide biomass measurements of dietary taxa [15,18]. At best, a semi-quantitative prediction of biomass consumed can be analysed from calculating the number of samples which contain a given food item (FOO), or from calculating the relative frequencies of sequence reads, coined relative read abundance (RRA) [15]. The RRA methodology is based upon the assumption that the number of sequences generated for a particular dietary taxon is proportional to the relative biomass of the dietary taxon consumed [82,83]. This method is not without caveats, as a recent meta-analysis by Lamb *et al.* [18] showed RRA and ingested food biomass showed a positive correlation in some model systems [32,84], but the same relationship was not found in others [85–87]. Taking these caveats into consideration, RRA methodology is not yet suitable for dietary quantification of highly generalist species such as hawfinch, which have the potential to consume a high number of different species. The use of FOO as a measure of importance can, however, conceal the true biological importance to the consumer [15]. This is due to all taxa given equal weight independent of the volume consumed, resulting in the importance of food taxa taken frequently in small amounts being artificially inflated within the dataset [15].

To conclude, hawfinch populations in the UK show dietary selectivity for resources previously observed to be highly used [44] and frequently occurring within their diet. This may be due to the net energy benefit gained by hawfinch from consuming these resources, the presence of secondary compounds which limits consumption of certain resources, or the seasonal switching of diet to match changing nutritional requirements. Whether the dietary selection found within this study directly translates to dietary importance is determined by the tree tissue type consumed and its nutritional value. While this was not investigated in this study, it is encouraged for future research. Having nutritional information will further knowledge regarding how sensitive hawfinch is to environmental changes, such as climate change or changes in woodland composition, factors which have been investigated as possible drivers of woodland bird decline [88].

The combined use of DNA metabarcoding and tree composition data in this study has enabled the feeding selectivity of hawfinch to be analysed for the first time. The combination of in-depth dietary data using molecular methodologies and knowledge of feeding selection can result in more in-depth analyses of woodland bird species diets and trophic interactions, leading to improved understanding of how woodland bird species are interacting within their environment. This has the potential to enhance understanding of the drivers behind the decline of woodland bird species.

## Data Availability

The data and code that support the findings of this study are openly available from the Dryad Digital Repository: https://doi.org/10.5061/dryad.h9w0vt4nh [89]. The data are provided in the electronic supplementary material [90].
